# Centrosome-phagy: implications for human diseases

**DOI:** 10.1186/s13578-021-00557-w

**Published:** 2021-03-04

**Authors:** Qi Wu, Xin Yu, Le Liu, Shengrong Sun, Si Sun

**Affiliations:** 1 Department of Breast and Thyroid Surgery, Renmin Hospital of Wuhan University, 238 Ziyang Road, Wuhan, 430060 Hubei People’s Republic of China; 2 Center of Ultramicroscopic Pathology, Renmin Hospital of Wuhan University, Wuhan, Hubei People’s Republic of China; 3 Department of Clinical Laboratory, Renmin Hospital of Wuhan University, 238 Ziyang Road, Wuhan, 430060 Hubei People’s Republic of China

**Keywords:** Centrosome, Autophagy, Ciliopathies, Aging, Cancer

## Abstract

Autophagy is a prominent mechanism to preserve homeostasis and the response to intracellular or extracellular stress. Autophagic degradation can be selectively targeted to dysfunctional subcellular compartments. Centrosome homeostasis is pivotal for healthy proliferating cells, but centrosome aberration is a hallmark of diverse human disorders. Recently, a process called centrosome-phagy has been identified. The process involves a panel of centrosomal proteins and centrosome-related pathways that mediate the specific degradation of centrosomal components via the autophagic machinery. Although autophagy normally mediates centrosome homeostasis, autophagy defects facilitate ageing and multiple human diseases, such as ciliopathies and cancer, which benefit from centrosome aberration. Here, we discuss the molecular systems that trigger centrosome-phagy and its role in human disorders.

## Centrosome composition and duplication

**Fig. 1:**
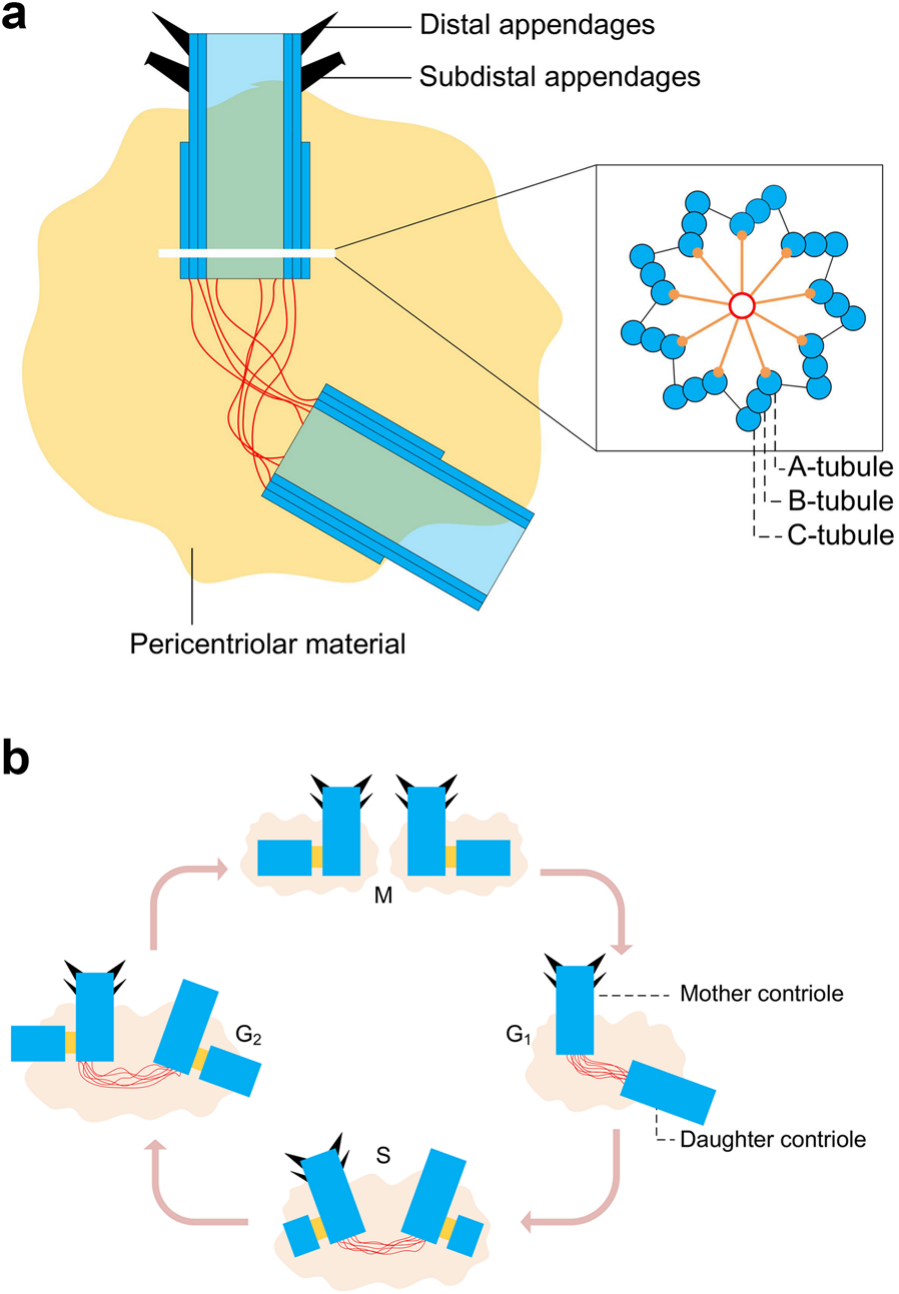
Centrosome composition and duplication. **a** The centrosome is composed of a pair of centrioles, which consist of fibres connecting their proximal ends and PCM surrounding the centriole pair. Nine microtubule triplets, dubbed the A-, B-, and C-microtubules within each triplet, are present in the proximal region of centrioles. The C-microtubule is absent from the distal region. In the proximal region, the A-microtubule and the C-microtubule are connected by an A–C linker. Other striking features present in mature centrioles are the subdistal and distal appendages; **b** Cells in the G1 phase contain two centrioles termed the mother and daughter centrioles. Towards the G1-to-S transition, a procentriole starts to assemble. During the S and G2 phases, each procentriole remains engaged in this configuration with its parental centriole and elongates. Towards the end of G2, the flexible link between the two parental centrioles is severed, allowing the two centrosomes to separate and govern assembly of the bipolar mitotic spindle

The centrosome is an evolutionarily conserved cylindrical organelle normally localized around the nuclei. It is composed of a pair of centrioles, which consist of fibres connecting their proximal ends and an amorphous cloud of different proteins surrounding the centriole pair called pericentriolar material (Fig. 1a) (PCM) [1]. A signature feature of the centriole is that nine sets of microtubules are arranged in a radially symmetrical manner at the organelle periphery [2]. The centriole is a polarized entity, with proximal and distal regions that differ notably in the number of microtubules they harbour. Thus, nine microtubule triplets, dubbed the A-, B-, and C-microtubules within each triplet, are present in the proximal region. The C-microtubule is absent from the distal region, so nine sets of microtubule doublets are present there. Furthermore, in the proximal region, the A-microtubule of one triplet and the C-microtubule of the adjacent triplet are connected by an A–C linker [3]. Other striking features present in mature centrioles are the subdistal and distal appendages. The latter are essential for docking beneath the plasma membrane upon templating the ciliary or flagellar axoneme [4]. Outside the centriole microtubule wall lies a cloud of proteins collectively forming the PCM, which is critical for the nucleation of cytoplasmic microtubules [5]. Another striking nine-fold symmetrical structure is the cartwheel present in the proximal-most ~ 100 nm of the procentriole, which elongate and develop as the new daughter centriole prior to mitosis [6]. The cartwheel can be observed before centriole microtubules during the assembly process in some systems and is essential for centriole biogenesis in most organisms [6], suggesting that it may impart the signature nine-fold radial symmetry to the entire organelle. The PCM supplies sites for microtubule nucleation, thus determining the number and composition of microtubules during the cell cycle. Therefore, all microtubule-related functions, including cell division, cell shape, polarity, motility and adhesion, are coordinated by centrosomes [7].

As with deoxyribonucleic acid (DNA) replication, the duplication of the centrosome is semi-conservative and is coupled with cell cycle progression in most cases, thus ensuring that each cell is endowed with the accurate complement of chromosomes and the correct number of two centrioles (Fig. 1b) [8]. During this process, the size of the centrosome is strictly regulated. Cells in the G1 phase contain two centrioles termed the mother and daughter centrioles, which are linked through a flexible structure connecting their proximal ends [9]. Towards the G1-to-S transition, a procentriole starts to assemble ~ 100 nm away from the microtubule wall of the proximal region of each parental centriole, almost orthogonally to the wall. During the S and G2 phases, each procentriole remains engaged in this configuration with its parental centriole and elongates to ~ 400 nm. Towards the end of G2, the flexible link between the two parental centrioles is severed, allowing the two centrosomes, each with a procentriole/centriole pair, to separate and govern assembly of the bipolar mitotic spindle [10]. During mitosis, each procentriole disengages from its parental centriole and loses the cartwheel, which is likely targeted for degradation. The two resulting centriolar cylinders in each daughter cell are then linked through their proximal ends via a flexible structure, thus completing the duplication cycle [9]. Together, these observations suggest that, in proliferating cells, the parental centriole provides a preferential scaffold that somehow favours the local assembly of a single and correct procentriole, thus ensuring faithful centriole duplication [10].

## Centrosome-phagy contributes to centrosome homeostasis

Centrosome homeostasis not only ensures the normality of centrosome number and structure, but also maintains the normal assembly process and function of centrosome, which is an essential factor for preserving cellular homeostasis and preventing disease onset [11]. And the deregulation of centrosome homeostasis is a hallmark feature of many human diseases [12]. Centrosome homeostasis is affected by many factors. For example, nuclear pore complex (NPC)-associated proteins Nup133 and Nup358 (also known as RanBP2) are involved in the control of centrosome position, and their absence lead to the failure of centrosome tethering to the nucleus at the G2/M transition, which is required for timely establishment of a properly positioned mitotic spindle [13, 14]. Overexpression or underexpression of some centrosome proteins like Polo-like kinase 4 (PLK4) and centrosomal P4.1 associated protein (CPAP, also known as Cenp-J) contribute to changes in the structure and quantity of centrosomes [15, 16]. And electron transport chain dysfunction (mitochondrial DNA depletion or electron transport chain inhibition) [17], failure of cytokinesis, mitotic slippage, cell–cell fusion and excessive centrosome duplication can lead to centrosome amplification [18]. In addition, recent studies have shown that autophagy is involved in regulating centrosome homeostasis [19–21].

**Fig. 2:**
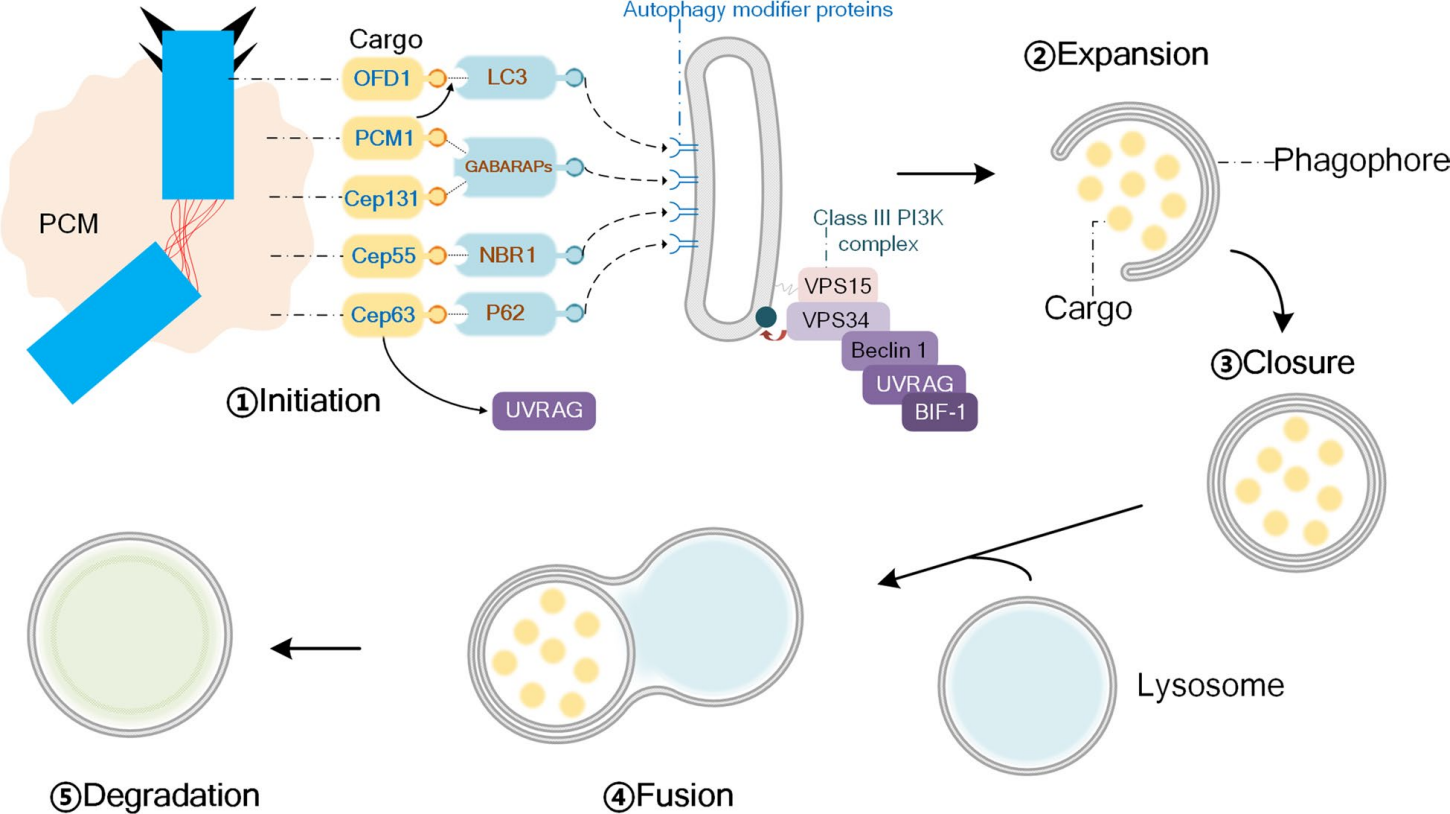
The process of centrosome-phagy. Specific autophagy receptor proteins recognize cargo proteins on the centrosome and are linked to autophagy-modifying proteins on the phagophore, which in turn trigger subsequent centrosome-phagy

Macroautophagy (hereafter referred to as autophagy) is a catabolic process. The formation of a double membrane phagophore (also known as an isolation membrane) is the key to the process of autophagy. This membrane forms an autophagosome after it elongates and closes, engulfs cellular material and transports it to lysosomes, and eventually the contents of the autophagosome are degraded and recycled [22]. Autophagosome formation is initiated by the Unc-51 like autophagy activating kinase (ULK) protein kinase complex [23]. The ULK protein kinase complex phosphorylates and activates the autophagy-related protein 14 (ATG14)-Beclin1-phosphatidylinositol 3-phosphate (PI3P) kinase complex, which resulting in a PI3P pool at autophagosome formation sites on the endoplasmic reticulum (ER) and recruited PICP-binding effectors double FYVE-containing protein 1 (DFCP1) and WD repeat domain phosphoinositide-interacting protein (WIPI) proteins. ATG12–5-16L1 complex is recruited to the phagophore membrane by WIPI2b, and it mediates the lipidation of cytosolic ATG8 proteins by the lipid phosphatidylethanolamine and membrane association [24]. Mammalian ATG8 proteins comprise two subfamilies, namely light chain 3 s (LC3s, including LC3A, LC3B and LC3C), and gamma-aminobutyric acid receptor-associated protein (GABARAPs, including GABARAP, GABARAP-like 1 and GABARAP-like 2), and play a crucial role in the formation and closure of the phagophore and the fusion of autophagosome and lysosome [25, 26]. Autophagosomes will be reduced in size and can not fuse with lysosomes after knocking out LC3s or GABARAPs. Moreover, the depletion of GABARAP inhibits the autophagy flux induced by starvation, and the depletion of LC3 leads to a decrease in cellular basal autophagy (autophagy level under non-induced conditions) [27]. Besides, the interaction between GABARAP and ATG2A/ATG2B is essential for phagophore closure [28]. Originally, autophagy was considered a non-selective process. However, increasing evidence suggests that there is another alternative type of autophagy called chaperone-mediated autophagy (CMA) that can deliver specific targets (such as proteins and organelles) to autolysosomes, leading to the degradion of these targets [22]. Specific autophagy receptors (such as P62, neighbor of BRCA1 gene 1 protein (NBR1)) and LC3s/GABARAPs are essential in this process. Autophagy receptors bind to cargo for degradation, and to LC3s/GABARAPs on the autophagosome membrane through the LC3-interacting region (LIR) [22]. Therefore, the genetic inactivation of autophagy receptors will cause the target protein or organelle to be unable to turnover without affecting other forms of selective or non-selective autophagy [22]. Evidence showed that loss of Beclin 1, a component of the autophagy machinery, increased the frequency of centrosome abnormalities, including increased centrosome number, indicating that autophagy plays a crucial role in maintaining the stability of centrosome [19]. Similarly, centrosome amplification disorganized autophagosome trafficking to lysosomes, resulting in an accumulation of autophagosomes [20], suggesting that autophagic flux was suppressed under centrosome-amplified conditions to prevent extra centrosomes from being degraded. Therefore, the concept of centrosome-phagy is proposed to describe the process of consumption of centrosome or centrosome components by selective autophagy. Nevertheless, the potential mechanism of centrosome-phagy has been described with the identification of the receptors needed for targeting centrosome fragments to the lysosome via a classical autophagy pathway (Fig. 2). To date, several centrosome-resident proteins have been identified to be degraded via selective centrosome-phagy or regulate centrosome-phagy, and multiple pathways connect autophagy and centrosome homeostasis.

## Centrosome-resident proteins as receptors for selective centrosome-phagy

### Cep63

Centrosomal protein 63 (Cep63) was first identified as a centrosome protein component by proteomic analysis of the centrosome and has been shown to function in the initial step of centriole duplication [29, 30]. Cep63 regulates mother-centriole-dependent centriole duplication by binding to Cep152 and then recruiting PLK4 to activate centriole biogenesis [29]. Likewise, Cep63 and Cep152 collaborate to promote the accumulation of essential centriole duplication factors upstream of SAS-6 (Spindle assembly abnormal protein 6 homolog) recruitment and procentriole formation to ensure efficient centriole duplication [30]. In this model, Cep192 is essential for the recruitment of Cep63 and Cep152 to the centrosome. Subsequently, knockdown of Cep63 and Cep152 could abolish homodimerized SAS-6 recruitment to the centrosome [30]. SAS-6 is recruited to the centrosome in S phase and forms the cartwheel structure based on the procentriole to act in centriole biogenesis [31]. Hence, Cep63 plays a valuable role in the regulation of centriole duplication.

A recent study indicated that Cep63 could be degraded by autophagy to regulate the number of centrosomes [32]. Moreover, the increase in Cep63 dots and multiple centrosomes found in p62^−/−^ mouse embryonic fibroblasts (MEFs) indicates a direct interaction between Cep63 and p62 (an adaptor or cargo receptor for autophagic degradation). Ultimately, autophagy engulfs and digests Cep63 dots to maintain centrosome homeostasis [32]. In addition, Cep63 is required for the centrosome localization of UV-irradiation-resistance-associated gene (UVRAG) [33]. UVRAG, as an autophagic initiator, primarily functions via interaction with Beclin 1 to activate the autophagy-related class III phosphoinositide 3-kinase (PI3K) complex [34]. Furthermore, disruption of the association between UVRAG and centrosomes leads to centrosome abnormalities and aneuploidy in a manner independent of its role in autophagy signalling [33]. Although there is no evidence at the present moment, the autophagy-mediated degradation of Cep63 may also maintain the centrosome homeostasis by regulating the autophagy-independent function of UVRAG. Given the central role of UVRAG in centrosome and autophagic clearing, its effect merits further exploration.

### PCM1

PCM1 has been identified as a centrosomal protein and provides a structural scaffold for the assembly of centriolar satellites (CSs) [21]. PCM1 is a large (~ 230 kDa) coiled-coil-containing protein. It can self-oligomerize and bind other CS proteins, such as the E3 ligase mind bomb 1 (MIB1) [35] and the deubiquitinase Ubiquitin Specific Peptidase 9 X-Linked (USP9X) [36]. There are different CS populations that contain different proteins; they colocalize with and bind to PCM1 and require PCM1 for their pericentrosomal localization. Moreover, PCM1 appears to regulate the actin-related proteins 2/3 (Arp2/3) complex and Wiskott–Aldrich syndrome protein (WASP) and Scar homologue (WASH) recruitment to the centrosome to mediate the centrosomal actin network [37].

Recently Holdgaard et al. identified PCM1-mediated selective autophagy of CSs and named it doryphagy [38, 39]. Interestingly, PCM1 interacts with GABARAPs but not LC3 and is then degraded by autophagy. GABARAPs are located on the pericentriolar matrix, and this dynamic pool contributes to autophagosome formation [39]. Previously, the PCM1 LIR motif was shown to be required for PCM1 colocalization with autophagosomes via direct interaction with GABARAPs [40]. This process does not depend on MIB1-mediated ubiquitylation [41]. Furthermore, PCM1 increases the formation and flux of GABARAPs/WIPI2/p62-positive autophagosomes without affecting LC3B-positive autophagosome formation [40].

Moreover, the study of Tang et al. showed that the interaction between LC3 and OFD1 (oral-facial-digital syndrome 1) was enhanced by PCM1 through autophagic processes to stimulate rapid degradation of OFD1 in MEFs [42]. OFD1 is the gene underlying the human disease oral-facial-digital syndrome type 1 (OFD1). An X-linked ciliopathy characterized by morphological abnormalities and renal cysts, as well as Joubert syndrome and Simpson–Golabi–Behmel syndrome type 2, can be caused by OFD1 abnormalities [43, 44]. OFD1 is located at the distal end of the centriole and the central granular satellite, so it is required for the formation of distal attachments, intraflagellar transport protein 88 (IFT88) recruitment and primary cilia formation and interacts with the human ciliary body disease-related proteins PCM1, Cep290, and Bardet–Biedl syndrome 4 (BBS4)[45]. Therefore, PCM1 is viewed as a specific CS receptor, and it is selectively recognized and degraded through autophagy to maintain centrosome integrity and accurate mitosis.

### Cep131 and Cep55

There are some other structural proteins of the centrosome involved in centrosome-phagy. First, Cep131 (~ 131 kDa, also called AZI1) is an evolutionarily conserved centriolar satellite protein associated with genomic stability maintenance and cilia formation [46, 47]. As a novel substrate of PLK4, Cep131 is phosphorylated to facilitate recruitment of SCL-interrupting locus protein (STIL) to the centriole, leading to centrosome amplification and cancer development [48]. Intriguingly, CEP131 is an ubiquitinated protein, and Cep131 stabilized by the deubiquitinase activity of USP9X promotes centrosome biogenesis and breast carcinogenesis [49]. Recently, Cep131 was shown to associate with LC3 in U2OS cells, as demonstrated by immunoprecipitation [42], suggesting that Cep131 may participate in the autophagic response. Likewise, Cep131 also interacted with GABARAPs but not LC3 in ATG8-inducible MCF7 cells, and bafilomycin A1, an autophagic inhibitor, led to the accumulation of Cep131, indicating that Cep131 is also degraded by autophagy under autophagy-inducing conditions [41]. Consistently, Cep55 localizes to the centrosome in interphase cells and is recruited to the midbody during cytokinesis. Mechanistically, Cep55 binds to the autophagic receptor NBR1 and is further tethered to the site of autophagosomal engulfment [50].

## The proteins as regulators for selective centrosome-phagy

### PLK1

Polo-like kinase 1 (PLK1) is the most frequently investigated PLK protein and has multiple effects on the cell cycle: coordinates the centrosome and cell cycles, controls mitotic initiation and the G2/M checkpoint, facilitates DNA replication, and plays multiple roles in spindle assembly and chromosome segregation [51].

It is worth considering that current research shows that PLK1 has a paradoxical role in autophagy modulation. For instance, PLK1 has been confirmed to induce autophagy in multiple cells. PLK1 is upregulated by natural neuroprotective autophagy enhancer corynoxine (Cory) in N2a neuroblastoma cell. In addition, Cory downregulated ribosomal protein S6 kinase and polypeptide 1 (p70S6K) to enhance autophagy, and the effect was dramatically diminished by inhibiting PLK1 [52]. Furthermore, Ruf et al. used Hela cells to demonstrate that PLK1 directly phosphorylates the component regulatory-associated protein of mTOR (RAPTOR, also known as RPTOR) in mechanistic target of rapamycin complex 1 (mTORC1) at lysosomes and inhibits the activity of mTORC1, thereby activating autophagy [53]. Hence, overactivation of PLK1 stimulates the autophagic response by decreasing the function of mTORC1 at lysosomes. In contrast, the PLK1 pharmacological inhibitors RO3280 and BI2536 induce autophagy to reduce tumour growth in androgen-insensitive (AI) castration-resistant prostate cancer (PCa) cells LNCaP-AI and acute promyelocytic leukemia NB4 cells [54, 55]. On the mechanism, both inhibitors contribute to the dephosphorylation of mTOR, which further increases the autophagic response.

**Fig. 3:**
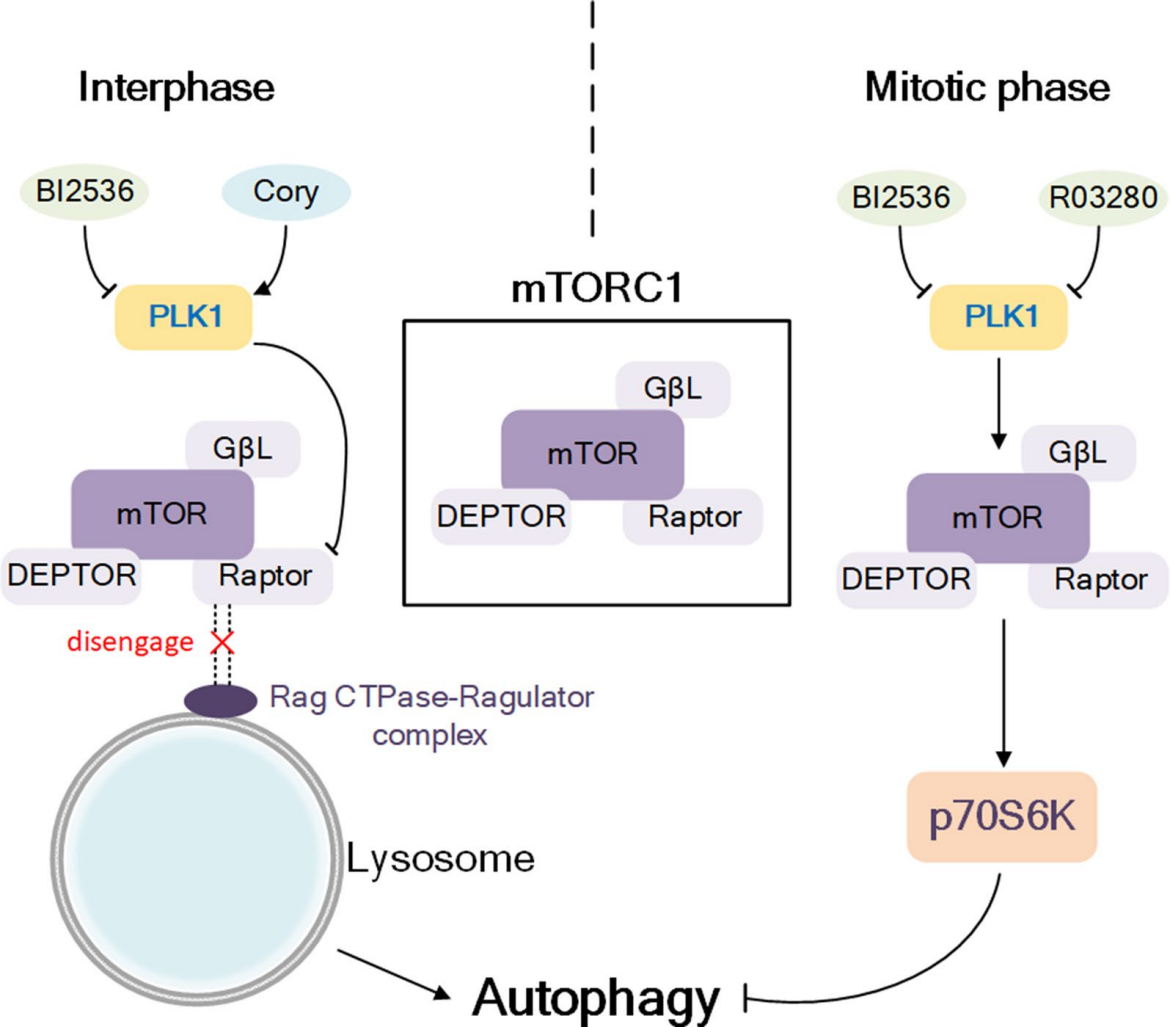
Different effects of PLK1 on autophagy in interphase and mitotic phase. In interphase, PLK1 can promote autophagy by phosphorylating Raptor to cause mTORC1 to disengage from lysosome. In mitotic phase, PLK1 promotes mTORC1 leading to up-regulation of p70S6K expression, thereby inhibiting autophagy

The potential reason for these controversial results may be the differences in different cell types or in the length of cell cycle arrest (Fig. 3). In the study of Ruf et al., PLK1 was inhibited by BI2536 in the short term (30 min) and combined with amino acid starvation [53]. Therefore, it mainly affects cells in interphase. During mitotic interval, PLK1 binds to mTOR and localizes on the surface of lysosomes, so overactivation of PLK1 causes the mTORC1 complex to disengage from lysosomes and thus activate autophagy. In the studies of Deeraksa et al. and Tao et al., PLK1 was inhibited in the long term (5 days and 8 days, respectively) [54, 55], which also inhibited PLK1 during mitosis. Based on the above data, we speculated that when cells are in mitotic phase, PLK1 is mainly located in the centrosome to ensure normal replication and division of the centrosome during mitosis. Inhibition of PLK1 will lead to centrosome abnormalities and mitotic errors, which further result in abnormal transcriptional pressure and protein aggregation, and activate autophagy-dependent degradation processes. The complex roles of PLK1 in autophagy regulation require further investigation.

### p53

The p53 tumour suppressor is considered the “guardian of ploidy”, acting in the prevention of centrosome structural or quantitative abnormalities through its transcriptional function [56–59]. However, the role of P53 located at the centrosome is still unclear. A previous study showed that P53 accumulates in centrosomes with simultaneous phosphorylation of Ser15 when TIG-1 human fibroblasts undergo cellular aging induced by serial passaging or oxidative stress [60]. Meanwhile, ataxia telangiectasia mutated (ATM) phosphorylates p53 Ser15 on discrete cytoplasmic p53 foci located at the centrosome of normal human lymphoblastoid cells (AHH1) [61].

**Fig. 4:**
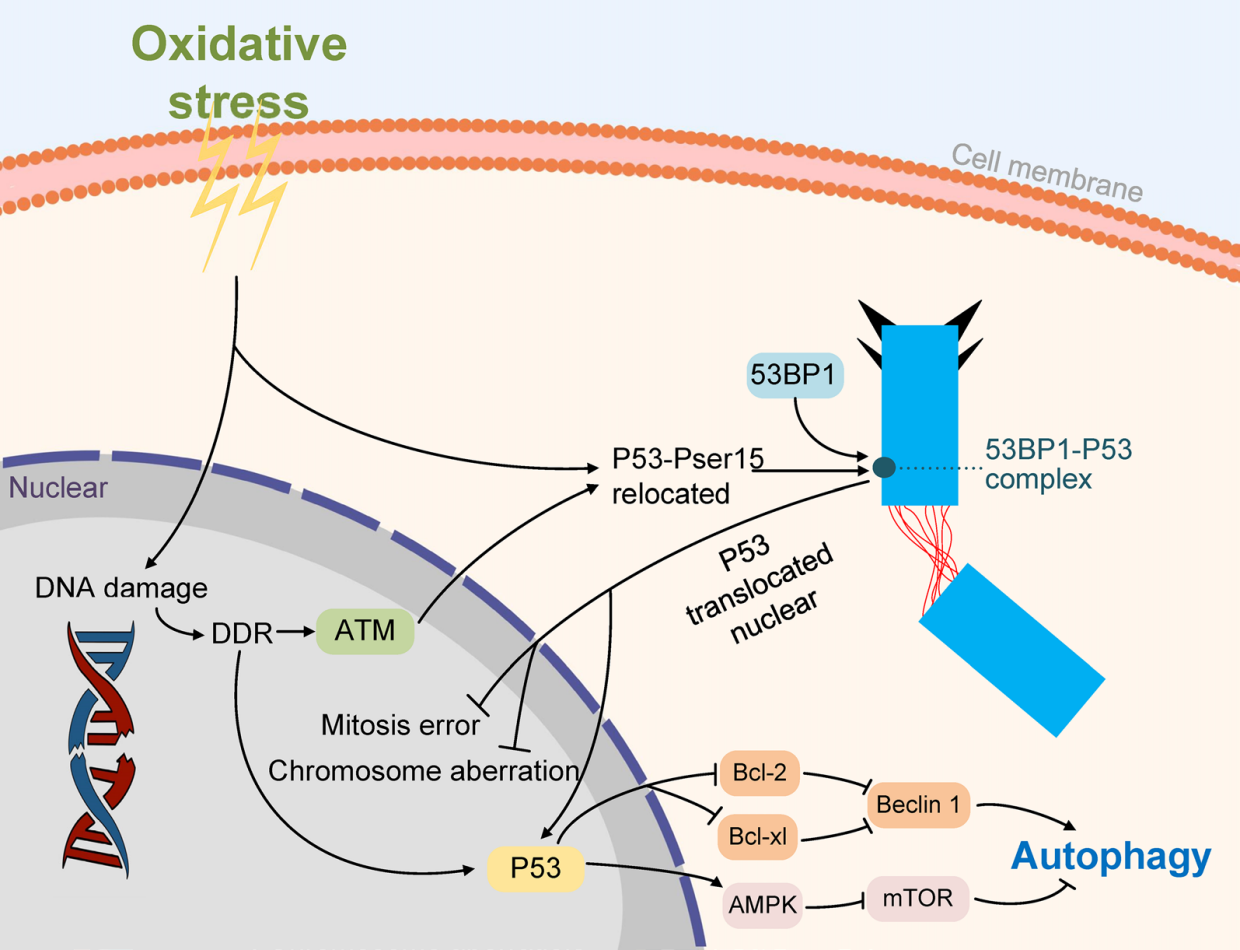
The function of p53 centrosome localization and the effect of p53 on autophagy. Under oxidative stress, P53 accumulates in the centrosome and binds to 53BP1 to form a complex to stabilize the structure of the centrosome and ensure its normal function. Under strong external stimulation conditions, the P53-53BP1 complex dissociates and P53 nuclear translocation will be induced to stimulate autophagy to maintain centrosome homeostasis and prevent mitosis error and chromosome aberration

It has long been known that p53 is located on centrosomes, but the function of p53 in this organelle is poorly understood. Recent studies have shown that selective impairment of p53 located at the centrosome can lead to centrosome fragmentation (Fig. 4). The essential effector p53-binding protein 1 (53BP1) in the mitotic surveillance pathway will bind to centrosomal P53, and the mitotic surveillance pathway will prevent human cells from growing under conditions with a high risk of making mitotic errors or accumulating numerous chromosome defects [62]. Evidence shows that induction of P53 and G1 arrest after centrosome depletion will be impaired by as deletion of either of 53BP1 or deubiquitinase ubiquitin-specific protease 28 (USP28), which are both essential components acting upstream of P53. 53BP1 is recruited to P53 through the BRCA1 C terminus (BRCT) domain, and the interaction between the BRCT domains of the 53BP1 and P53 is necessary for centrosome monitoring pathways other than normal DNA damage response (DDR) [63]. The main function of P53-53BP1 complex centrosome localization is to stabilize the structure of centrosome and ensure its normal function. Moreover, under conditions of strong external stimuli such that P53-53BP1 complex cannot guarantee the stability of centrosome structure, nuclear translocation of P53 will be induced to stimulate autophagic activity, and then degrade abnormal centrosome proteins to ensure the centrosome homeostasis [60]. In addition, p53 is demonstrated to guarantee correct spindle pole positioning and chromosome segregation by stimulating centrosome separation [59]. Ultimately, overexpression of Cep55 is discovered in the majority of human cancers with inactivation of p53. p53 reduces the protein stability of Cep55 and then downregulates the expression of Cep55 by inhibiting the activity of PLK1 [57]. Thus, it is possible that the phosphorylation and accumulation of p53 in the centrosome is the pivotal event that guarantees the normal function of the centrosome and exerts an anti-tumour effect. Importantly, p53 also functions as a well-studied connector linking autophagy and stress-induced cell cycle responses. After DNA damage, nuclear p53 activates various autophagy-related signalling pathways to induce autophagy, including activating the AMP-activated protein kinase (AMPK)-mTOR pathway and releasing Beclin 1 from inhibitory interactions with B-cell lymphoma-2 (Bcl-2) and B-cell lymphoma-extra large **(**Bcl-XL) [64]. p53 induces autophagy to remove abnormal centrosome proteins, which may be a way to ensure normal mitosis of cells. Therefore, further research is needed to understand whether centrosomal p53 induces autophagy and how this process is regulated.

## Insights into centrosome-phagy-related human disorders

Centrosome defects can be roughly divided into numerical abnormalities and structural abnormalities, of which structural abnormalities include centriole structural defects and PCM component defects [12]. As mentioned above, centrosome-phagy regulates the numerical abnormalities and structural abnormalities of the centrosome by degrading the centrosome or specific centrosome components. Based on the complexity and importance of centrosome function [65], centrosome-phagy is associated with various human disorders, including ciliopathies, ageing and cancer (Table 1).

**Table 1:** Human disorders linked to centrosome-phagy

Centrosome-phagy-associated human disorders		Relevant proteins
Ciliopathies	Polycystic kidney disease, retinitis pigmentosa, Joubert syndrome	OFD1 [98–100]
	Polycystic kidney disease, Bardet–Biedl syndrome, Joubert syndrome, Meckel syndrome	PCM1 [69–71, 101]
Ageing	Huntington disease	PCM1 [102]
Cancer	Thyroid, renal, breast, ovarian and colon cancer	Cep55 [57, 95, 103–105]
	Neuroblastoma	Cep63 [97]
	Breast cancer	Cep131 [49]

### Ciliopathies

The centrosome is closely related to ciliogenesis. Similar to centriole, cilia are nine-fold symmetrical microtubule-based cylindrical structures and extend from the surface of the cell [66]. Ciliogenesis mainly occurs in the G1-S phase, when the mother centriole matures into basal body and migrates to the cell surface. Then the distal appendages of the basal body directly interacts with the plasma membrane to form the ciliary membrane, and the doublet microtubules of basal body began to elongate to form axons extending cell periphery to form the cilia [67]. Thus, centrosome defects can lead to]disorders of ciliogenesis, which contributes to diverse developmental and degenerative disorders categorized as ciliopathies, which include polycystic kidney disease (PKD), nephronophthisis, retinitis pigmentosa, Bardet–Biedl syndrome, Joubert syndrome, and Meckel syndrome [68].

Evidences suggest that both mislocalization and depletion of PCM1 can lead to suppression of ciliogenesis. Mutation or knockdown of BBS4 can cause PCM1 to be mislocalized, thereby inhibiting ciliogenesis in vivo and in vitro [69, 70]. And mutation of Cep290, which is the inducer of Joubert syndrome and associated with Meckel syndrome and Bardet–Biedl syndrome, depletes PCM1 from the centrosome to reduces ciliogenesis [71]. Likewise, autophagy regulates ciliogenesis by removing cilia-related proteins to ensure the proper length of cilia. This phenomenon suggest the potential for modulation of autophagy as a new therapeutic opportunity in ciliogenesis-related diseases [72]. As previously mentioned, the ciliopathy-related protein OFD1 located in centriolar satellites is degraded via autophagy to promote primary cilium biogenesis in MEFs and retinal pigment epithelium (RPE) cells [42]. PKD is a representative ciliopathy caused by mutations in the PKD1 or PKD2 gene [73]. Evidences show that congenital PKD mice are defective in autophagy. Meanwhile, suppression of autophagy is associated with defective cilia, and various autophagy-inducing agents could protect against PKD [73]. These evidences further indicate that autophagy is involved in cilia formation.

In summary, centrosome-phagy may potentially contribute to the ability of centriolar satellites to exert proper function. However, the detailed mechanisms by which inhibited autophagy is associated with decreased autophagy in diverse ciliopathies and conditions with defective centrosomes remain unclear.

### Ageing

Ageing is a natural biological process of all living organisms, the characteristics of which include disruptions in cellular metabolism and function that change with time and lead to permanent cell cycle arrest and cell death [74].

Direct evidence supports a causal link between centrosome aberrations and ageing-related diseases [75]. The centrosomes are fragmented in cells undergoing replicative senescence or premature senescence induced by oxidative stress [76], accompanied by p53 centrosome localization and phosphorylation at Ser15 [60]. And centrosome fragmentation and the initiation of premature senescence can be caused by the disruption of core PCM components neural precursor cell expressed developmentally down-regulated protein 1 (NEDD1) and Cep192 [76]. Meanwhile, percentcentrin can be recruited to PCM by PCM1. Exit from the cell cycle can be induced by inhibition of percentenrin or PCM1, accompanied by increased expression of cellular β-galactosidase, a hallmark of cellular senescence [77]. These evidences indicating that the progression of cellular senescence can be promoted by centrosome defects. In addition, it has been established that autophagy-driven homeostatic restorations determine the lifespan of several model organisms. Evidences show that autophagy dysfunction occurs in ageing tissues and several aging-related diseases and decreased autophagy leads to accelerated aging process [78]. Meanwhile, organelle-initiated autophagy, including mitophagy (mitochondrial autophagy) [79], ER-phagy [80], centrosome-phagy, etc., also inhibit the aging process. Similarly, organelle-initiated autophagy can degrade damaged organelles or organelle components to maintain organelle functions. A few studies have demonstrated that suppression of mitophagy is exhibited in senescent cells and leads to a defective mitochondrial network that might result in metabolic dysfunction during aging [81, 82]. Specifically, Alzheimer’s disease and Parkinson’s disease are primary ageing-related diseases [83]. Defective autophagy supports a pathogenic role of protein aggregation (Alzheimer disease-associated β-amyloid and Parkinson disease-associated SNCA/α-synuclein), which is the main pathological feature in neurodegenerative diseases [84]. As mentioned above, Cory, as a neuroprotective autophagy enhancer, promoted the clearance of β-amyloid and α-synuclein (SNCA)/ by enhancing autophagy, and these effects dramatically rely on the centrosome-associated kinase PLK1 [52].

Therefore, it is speculated that centrosome-phagy defects contribute to the accumulation of centrosomal abnormalities to drive senescence. Furthermore, a potent therapeutic strategy that targets the connection between the centrosome and autophagy has the possibility to improve ageing-associated syndromes.

### Cancer

Centrosome aberrations are commonly observed in many different cancers, including breast, prostate, colon, ovarian and pancreatic cancer, and multiple myeloma, non-Hodgkin's and Hodgkin's lymphomas, acute and chronic myeloid leukaemia [85, 86]. The underlying molecular mechanisms of centrosome dysfunction and its effect on cancer have recently begun to be explored. First, abnormal centrosome structures are usually observed in cancer cells. Centriole size is usually increased and displays significant over-elongation in cancer cells. And the centriole over-elongation drives centriole amplification [87]. In addition, evidences show that larger centrosomes in cancer cells are associated with stronger aggressiveness [88]. Besides, the centrosome can be misshapen in the form of string-like elongated linear arrays, ring-like, amorphous, atypical filaments, and corkscrew in cancer cells. These structural defects in the centrosome can lead to the formation of false mitotic spindles, which contribute to chromosomal missegregation and aneuploidy [89]. Consistently, centrosome amplification lead to genome instability to cause tumorigenesis and is associated with high-grade tumors and a poor prognosis [85]. Moreover, centrosome amplification can enhance stem cell division in Drosophila without significantly affecting genetic stability [90]. Second, centrosome aberrations can also facilitate the dissemination of metastatic cells by disrupting tissue architecture and confer invasive properties. Recent studies have shown that extra centrosomes induce Ras-related C3 botulinum toxin substrate 1 (RAC1) activation by increasing microtubule nucleation and then promote tumour invasion through RAC1 [91]. The extra centrosome-associated secretory pathway (ECASP) has been considered to contribute to significant changes by inducing the release of a variety of pro-invasive factors (IL-8 and growth differentiation factor 15 (GDF-15)) that are associated with tumorigenesis and tumour metastasis [92]. Finally, evidences showed that centrosome defects can induce tumor drug resistance through multiple pathways [93]. Centrosome amplification is induced by PLK4 overexpression and promotes chromosomal instability, leading to breast cancer cells resistance to tamoxifen and trastuzumab [94]. Mitotic slippage, which is described as the process of cells leave mitosis without completing a normal cell division and become tetraploid, can be induced by overexpression of Cep55 and enhance breast cancer resistance to chemotherapy drugs, especially docetaxel [95]. In addition, the ATP-binding cassette (ABC) transporters members, as efflux drug pumps, can be activated to induce drug resistance by the overexpression of centrosome protein NIMA related kinase 2 (NEK2), including ABCB1 (p-glycoprotein, MDR1), the multidrug resistance protein ABCC1 (MRP1), and the breast cancer resistant protein ABCG2 (BCRP) [94, 96].

A strong connection between centrosome amplification and autophagy in cancer was recently demonstrated. Evidence suggests that overexpression of Cep63 in U2OS cells can lead to centrosome amplification and is also associated with aggressive malignancies [97]. As mentioned above, Cep63 can be degraded by autophagy to inhibit centrosome amplification [32]. Moreover, the results show that centrosome amplification causes an accumulation of autophagosomes by interfering with autophagic flux [20]. Meanwhile, selectively activated autophagy maintains chromosomal stability and centrosome number to suppress tumour progression [19]. Taken together, these findings show that centrosome abnormalities potentially inhibit the autophagic response to promote tumour progression. A novel precision medicine strategy targeting centrosome-phagy may be important for anti-tumour therapeutic exploitation.

## Conclusion and perspective

Understanding the effect of centrosome-phagy is an important research direction that has been widely ignored. Although there is little evidence, the available data strongly support the opinion that autophagy is associated with the function and integrity of the centrosome. Based on the fact that several centrosome phagocytic receptors have been identified and may be more commonly identified in the future, the analysis of their biological functions can be a challenging task. They may work collaboratively or have specific functions during centrosomal autophagy responses. For example, Cep63, as a valuable regulator of the initiation of centriole duplication, can be degraded in autophagosomes to maintain centrosome number, while PCM1, as the structural scaffold around the centriole, recruits GABARAP-associated autophagosomes to retain the normal structure of the centrosome. It is likely that different receptors may also mediate different pathways to activate autophagy. Cep63 directly binds to p62 and sustains the location of UVRAG at the centrosome; in contrast, PCM1 mainly interacts with GABARAPs via the PCM1 LIR motif.

Understanding the molecular pathways responsible for the activation of centrosome-phagy is also challenging. Currently, little is known about the basic molecular mechanisms of centrosome-phagy. The multitudinous kinases involved in the duplication and function of the centrosome are attractive as potential targets of therapy but require further study. For example, PLK1 not only plays an important role in the centrosome cycle but also stimulates the autophagic response by decreasing the function of mTORC1 at lysosomes. In addition, p53 may connect centrosome-phagy and specific stress stimuli. However, the detailed effect of p53 on centrosome-phagy is still an open question. We think that exploring answers to these questions will provoke an exciting surge in research involving the relationship between autophagy, autophagy-related signalling, and diverse stress stimuli, which are all closely associated with proper centrosome function.

The role of centrosome-phagy in the pathogenesis of human diseases remains to be seriously considered. Centrosome-phagy regulates the elimination of disease-related proteins, which is relevant to the pathogenesis of numerous ciliopathies. Moreover, centrosome-phagy may also be involved in ageing-related diseases, so it may affect neurological functions directly or indirectly involved in the development of neurodegenerative diseases. Ultimately, studies on the potential interactions between centrosomes and autophagy could better elucidate the impact of centrosome-phagy on tumour progression. Due to the widespread distribution of centrosome-phagy in human disease, the identification of therapies related to centrosome-phagy is pivotal.

## Data Availability

Not applicable.

## Abbreviations

53BP1: P53-binding protein 1
AI: Androgen-insensitive
AMPK: AMP-activated protein kinase
Arp2/3: Actin-related proteins 2/3
ATG: Autophagy-related protein
ATM: Ataxia telangiectasia mutated
BBS4: Bardet–Biedl syndrome 4
Bcl-2: B-cell lymphoma-2
Bcl-XL: B-cell lymphoma-extra large
BICD2: BICD cargo adaptor 2
BRCT: BRCA1 C terminus
Cep: Centrosomal protein
Cory: Corynoxine
CMA: Chaperone-mediated autophagy
CPAP: Centrosomal P4.1 associated protein
cryo-ET: Cryo-electron tomography
CSs: Centriolar satellites
DNA: Deoxyribonucleic acid
WIPI: Double FYVE-containing protein 1
DDR: DNA damage response
ECASP: Extra centrosome-associated secretory pathway
GABARAPs: Gamma-aminobutyric acid receptor-associated protein
GDF-15: Growth differentiation factor 15
LC3: Light chain 3
LIR: LC3-interacting region
IFT88: Intraflagellar transport protein 88
MEF: Mouse embryonic fibroblasts
MIB1: Mind bomb 1
mTORC1: Mechanistic target of rapamycin complex 1
NBR1: Neighbor of BRCA1 gene 1 protein
NEDD1: Neural precursor cell expressed developmentally down-regulated protein 1
OFD1: Oral-facial-digital syndrome type 1
p70S6K: Protein S6 kinase and polypeptide 1
PCa: Castration-resistant prostate cancer
PCM: Pericentriolar material
PI3K: Phosphoinositide 3-kinase
PKD: Polycystic kidney disease
PLK1: Polo-like kinase 1
PLK4: Polo-like kinase 4
RAC1: Ras-related C3 botulinum toxin substrate 1
RAPTOR: Regulatory-associated protein of mTOR
RPE: Retinal pigment epitheliums
SNCA: α-Synuclein
SAS-6: Spindle assembly abnormal protein 6 homolog
STIL: SCL-interrupting locus protein
ULK: Unc-51 like autophagy activating kinase
USP28: Ubiquitin-specific protease 28
USP9X: Ubiquitin specific peptidase 9 X-linked
UVRAG: UV-irradiation-resistance-associated gene
WASP: Wiskott–Aldrich syndrome protein
WASH: WASP and Scar homologue
WIPI2: WD repeat domain phosphoinositide-interacting protein 2
